# Electronic and Steric Effects in Gold(I) Phosphine Thiolate Complexes

**DOI:** 10.1155/MBD.1994.405

**Published:** 1994

**Authors:** Janet Foley, Raymond C. Fort, Katherine McDougal, Mitchell R. M. Bruce, Alice E. Bruce

**Affiliations:** 1 Department of Chemistry University of Maine Orono ME 04469-5706 USA

## Abstract

The unusual yellow color of Au_2_(dppm)(SR)_2_ (R = 4-tolyl; dppm = diphenylphosphinomethane) is attributed to a red-shift in the S→Au charge transfer caused by destabilization of the sulfur highest occupied molecular orbital (HOMO). Variable temperature experiments show two broad bands at -80°C in the ^31^P{^1^H} NMR spectrum of Au_2_(dppm)(SR)_2_ and the activation energy for interconversion is 10 kcal/mol. Only one sharp band is observed down to -80°C in the spectrum of the white complex, Au_2_(dppe)(SR)_2_ (dppe = diphenylphosphinoethane). Molecular mechanics calculations on Au_2_(dppm)(SR)_2_ and Au_2_(dppe)(SR)_2_ reveal that, for Au_2_(dppe)(SR)_2_, a series of maxima and minima, separated by 2.5 kcal/mol, occur every 120° which is consistent with rotation around an unhindered carbon-phosphorus single bond. The Au atoms are not within bonding distance in any conformation. Computational results for Au_2_(dppm)(SR)_2_ indicate one minimum energy structure in which the Au-P bonds are anti. There is a high energy conformation (9 kcal/mol above the global minimum) where overlap between golds is maximized. The implications of gold-gold bonding in this complex are discussed. The steric influence of the thiolate ligand has been examined by synthesizing a series of dinuclear gold(I) complexes in which the steric properties of the thiolate are varied: Au_2_(dppm)(SR)_2_ (R = 2,6-dichlorophenyl; 2,6-dimethylphenyl; 3,5-dimethylphenyl). The 2,6-disubstituted complexes are white, while the 3,5-dimethyl complex is yellow. These results, along with VT-NMR experiments, are consistent with the conclusion that the more sterically-bulky thiolates hinder the close approach of the golds in the dinuclear complexes.


### ELECTRONIC AND STERIC EFFECTS IN GOLD(I) PHOSPHINE THIOLATE COMPLEXES

Janet Foley, Raymond C. Fort, Jr., Katherine McDougal, Mitchell R. M. Bruce, and Alice E. Bruce*

Department of Chemistry, University of Maine, Orono, ME 04469-5706, USA

The unusual yellow color of $Au_2(dppm)(SR)_2$ (R = 4-tolyl; dppm = diphenylphosphinomethane) is attributed to a red-shift in the S→Au charge transfer caused by destabilization of the sulfur highest occupied molecular orbital (HOMO). Variable temperature experiments show two broad bands at -80°C in the $^{31}P\{^1H\}$ NMR spectrum of $Au_2(dppm)(SR)_2$ and the activation energy for interconversion is 10 kcal/mol. Only one sharp band is observed down to -80°C in the spectrum of the white complex, $Au_2(dppe)(SR)_2$ (dppe = diphenylphosphinoethane). Molecular mechanics calculations on $Au_2(dppm)(SR)_2$ and $Au_2(dppe)(SR)_2$ reveal that, for $Au_2(dppe)(SR)_2$, a series of maxima and minima, separated by 2.5 kcal/mol, occur every 120°. which is consistent with rotation around an unhindered carbon-phosphorus single bond. The Au atoms are not within bonding distance in any conformation. Computational results for $Au_2(dppm)(SR)_2$ indicate one minimum energy structure in which the Au-P bonds are anti. There is a high energy conformation (9 kcal/mol above the global minimum) where overlap between golds is maximized. The implications of gold-gold bonding in this complex are discussed. The steric influence of the thiolate ligand has been examined by synthesizing a series of dinuclear gold(I) complexes in which the steric properties of the thiolate are varied: $Au_2(dppm)(SR)_2$ (R = 2,6-dichlorophenyl; 2,6-dimethylphenyl; 3,5-dimethylphenyl). The 2,6-disubstituted complexes are white, while the 3,5-dimethyl complex is yellow. These results, along with VT-NMR experiments, are consistent with the conclusion that the more sterically-bulky thiolates hinder the close approach of the golds in the dinuclear complexes.

### Introduction

During the last decade, interest in gold chemistry has increased for several reasons, the most important one being that certain gold(I) thiolate compounds are very effective drugs for the treatment of rheumatoid arthritis, a poorly-understood disease that afflicts millions of people each year.[1] The therapeutic benefits of gold have also prompted testing of gold(I) phosphine and thiolate compounds for anticancer activity and inhibition of the HIV-1 virus.[2] In addition to interest in the biological activity of gold, the intriguing phenomenon of attractive interactions between closed shell gold(I) atoms ([$Xe4f^{14}5d^{10}$]), has stimulated numerous experimental and theoretical studies.[3] Material scientists have also long been interested in gold and in a recent report of the use of gold as a sensor for thiols, the authors commented on the need for more studies of the fundamental properties and reactivities of gold-thiolate compounds.[4]

Several years ago, our group initiated studies of the electronic structure and reactivity of neutral gold(I) phosphine thiolate complexes.[5] Our interest in these complexes is motivated by their similarity to Auranofin, an orally active anti-arthritis gold(I) drug that contains triethylphosphine and thiolate ligands. We recently reported the synthesis and characterization of several classes of dinuclear and mononuclear gold(I) complexes containing phosphine and thiolate ligands (series **A-C**, see below).[5a] All of the complexes in series A are white with the exception of **A1**, which is bright yellow, an atypical color for linear two-coordinate gold(I). The UV-vis spectra are similar for all complexes, consisting of intense absorbances above 40,000 $cm^{-1}$ (< 250 nm) followed by a series of poorly defined shoulders at lower energy. Spectral fitting procedures were used to deconvolute the spectra into a series of Gaussian bands (see Figure 1).[5a]

Classical techniques involving changing the solvent polarity and varying the ligand electronic properties were used to assign the two lowest energy transitions as sulfur-to-gold charge transfers

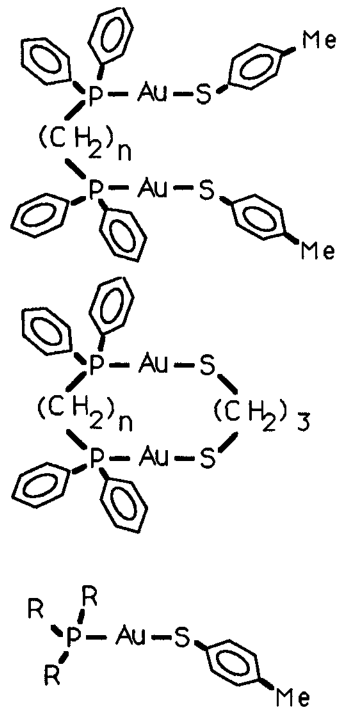

A

n = 1 2 3 4 5

A1 A2 A3 A4 A5

B

n = 2 3 4 5
B1 B2 B3 B4

C

R = Ph Me
C1 C2

(S→Au CT).[6] In all the complexes except **A1**, the S→Au CT's occur in the UV, whereas in **A1** the lowest energy band is red-shifted by 2500 $cm^{-1}$ into the visible, hence the yellow color of **A1** (see Table I). The red-shift could result from destabilization of the highest occupied molecular orbital

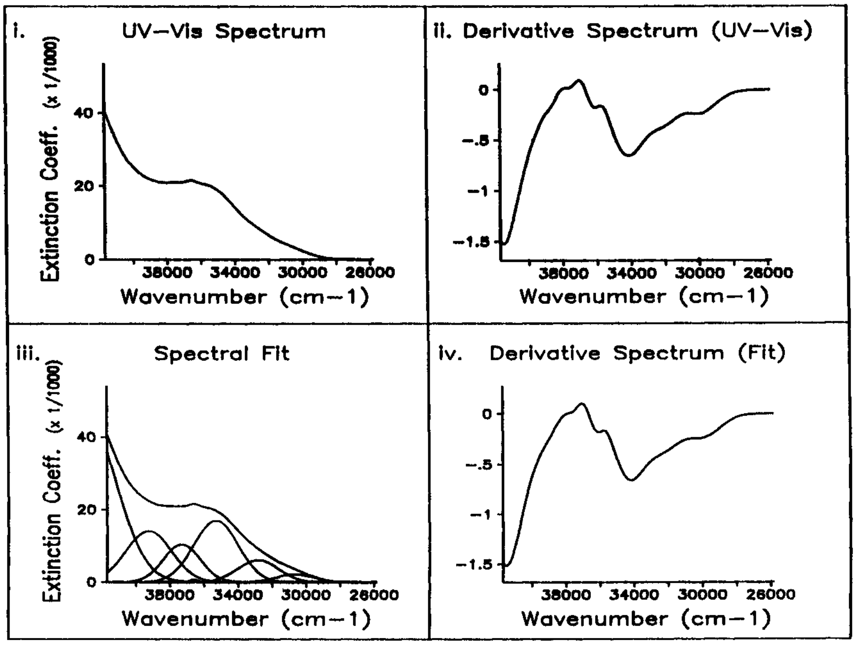

**Figure 1.** UV-visible absorption and derivative spectra for $[Au_2(dpppn)(p\text{-}tc)_2]$ (**A5**). (i) experimental absorption spectrum; (ii) experimental derivative spectrum; (iii) spectral fit for absorption spectrum; (iv) spectral fit for derivative. (Reprinted with permission from Narayanswamy, R; Young, M. A.; Parkhurst, E.; Ouellette, M.; Kerr, M. E.; Ho, D. M.; Elder, R. C.; Bruce, A. E.; Bruce, M. R. M. *Inorg. Chem.*, 1993, **32**, 2202. Copyright 1993 American Chemical Society.

(sulfur), stabilization of the lowest unoccupied molecular orbital (gold), or a combination of both. Variable temperature NMR experiments and molecular mechanics computations on **A1** and **A2** provide insight to the reason for the red shift in the UV-vis spectrum of **A1**.[7]

**Table I.** Electronic Absorption Spectral Features of selected Gold(I) Phosphine Thiolate Complexes. Comparison of Absorption Bands, $cm^{-1}$, and (extinction coefficients).

| $Au(PPh_3)(p\text{-tc})$ | $Au_2(dpppn)(p\text{-tc})_2$ | $Au_2(dppm)(p\text{-tc})_2$ | Assignment |
|---|---|---|---|
| $3.0 \times 10^4$ $(1 \times 10^3)$ | $3.05 \times 10^4$ $(2 \times 10^3)$ | $2.8 \times 10^4$ $(3 \times 10^3)$ | LMCT (S→Au) |
| $3.25 \times 10^4$ $(3 \times 10^3)$ | $3.3 \times 10^4$ $(6 \times 10^3)$ | $3.1 \times 10^4$ $(6 \times 10^3)$ | LMCT (S→Au) |
| $3.5 \times 10^4$ $(7 \times 10^3)$ | $3.55 \times 10^4$ $(2 \times 10^4)$ | $3.45 \times 10^4$ $(2 \times 10^4)$ | IL (*p*-tc) |
| $3.6 \times 10^4$ $(8 \times 10^2)$ | $3.65 \times 10^4$ $(7 \times 10^2)$ | $3.6 \times 10^4$ $(3 \times 10^2)$ | $\pi \rightarrow \pi^*$ |
| $3.7 \times 10^4$ $(8 \times 10^3)$ | $3.75 \times 10^4$ $(1 \times 10^4)$ | $3.7 \times 10^4$ $(1 \times 10^4)$ | $n \rightarrow \pi^*$ |
| $3.85 \times 10^4$ $(3 \times 10^3)$ | $3.9 \times 10^4$ $(1 \times 10^4)$ | $3.9 \times 10^4$ $(1 \times 10^4)$ | |

### Materials and Methods

**Reagents and Instrumental Details.** Bis(diphenylphosphino)methane and bis(diphenylphosphino)ethane were purchased from Aldrich or Strem and used as received. $HAuCl_4 \cdot 3H_2O$ was obtained from Aldrich or Aithaca. The thiols, *p*-thiocresol (4-methylthiophenol), 4-chlorothiophenol, 3,5-dimethylthiophenol, 2,6-dimethylthiophenol, and 2,6-dichlorothiophenol were purchased from Aldrich. Bis(*p*-chlorophenyl)disulfide was purchased from ChemService. Cambridge Isotope Labs supplied $CD_2Cl_2$ used for NMR experiments. The $^{31}P\{^1H\}$ NMR data were obtained on a Varian XL-200 FT-NMR spectrometer operating at 81 Mz and chemical shifts are referenced to external 85% $H_3PO_4$. Variable-temperature NMR experiments were carried out from +20 to -80 °C on 0.035 M and 0.07 M solutions of **A1** and ca. 0.07 M solutions of **A2** in $CD_2Cl_2$.

**Synthesis and Abbreviations.** Complexes **A1** and **A2** are prepared as air stable, microcrystalline solids by using procedures previously described.[5a] The complexes in series **D** are prepared in a similar fashion, by using thiolate substitution reactions of the corresponding dinuclear gold(I) phosphine chloride complexes. The following abbreviations are used: dppm = bis(diphenylphosphino)methane; dppe = 1,2-bis(diphenylphosphino)ethane; *p*-tc = *p*-thiocresol.

**Computational Methodology.** Molecular mechanics calculations were performed on a Gateway 200 486DX2 (66Mhz, 16Mb memory), employing the program PCModel.[8] PCModel contains the MMX force field, which incorporates both MM2[9] and MM3[10] parameterization, as well as additional parameters developed by Serena. Aromatic carbons (type 40) were used for the benzene rings; no π-VESCF calculations were performed. The program implements a set of generalized force constants for transition metal atoms, and uses standard covalent radii: for example, 1.455Å for gold. These parameters result in realistic geometries for simple coordination compounds; thus, a calculation on $[Au(SCH_3)_2]^-$ produces a linear geometry around gold. However PCModel contains no parameters for repulsions between transition metals. Standard bond lengths provided by the program were used (e.g. Au-P = 2.47Å and Au-S = 2.49Å) and the bond lengths were not fixed for the calculations reported here. Additional calculations carried out with bond lengths fixed at values more characteristic for Au(I)-P (2.26Å) and Au(I)-S (2.3Å) bonds, gave similar results, however the total energy was higher due to increased steric interactions. Complexes **A1** and **A2** were minimized at arbitrary geometries and the ROT E function, which utilizes the rigid rotor approximation, was employed to locate additional minima. Structures so located were subjected to full geometry optimization, including vibrational annealing at 300 K.

The dihedral driver of PCModel was used to calculate conformational energy profiles for each complex. The dihedral angles, $\phi_1$ and $\phi_2$ for **A1** and $\phi_3$ for **A2** are defined as illustrated in

Scheme I. Two dihedrals were defined for **A1** for the purpose of carrying out a double dihedral drive calculation. For single dihedral drive computations, a full geometry optimization was carried out for every 5° rotation in dihedral angle ($\phi_1$ or $\phi_3$) beginning at 0°. For the double dihedral drive computation, $\phi_2$ was rotated by 60° increments and a full geometry optimization was carried out for every 5° rotation in $\phi_1$ beginning at 0°. Distances between gold atoms were determined by using the QUERY command. There are discontinuities in the conformational energy profiles at the starting and ending dihedrals; e.g. at 0° and 360° in Figure 3. Discrepancies in energy of several kcal/mol for conformations at the starting and ending points of the dihedral drive computation are typical when those points lie in regions of relatively steep slope. Resetting initial and final points brings the results of different computations into agreement in these regions within 0.2 kcal/mol.

SCHEME I

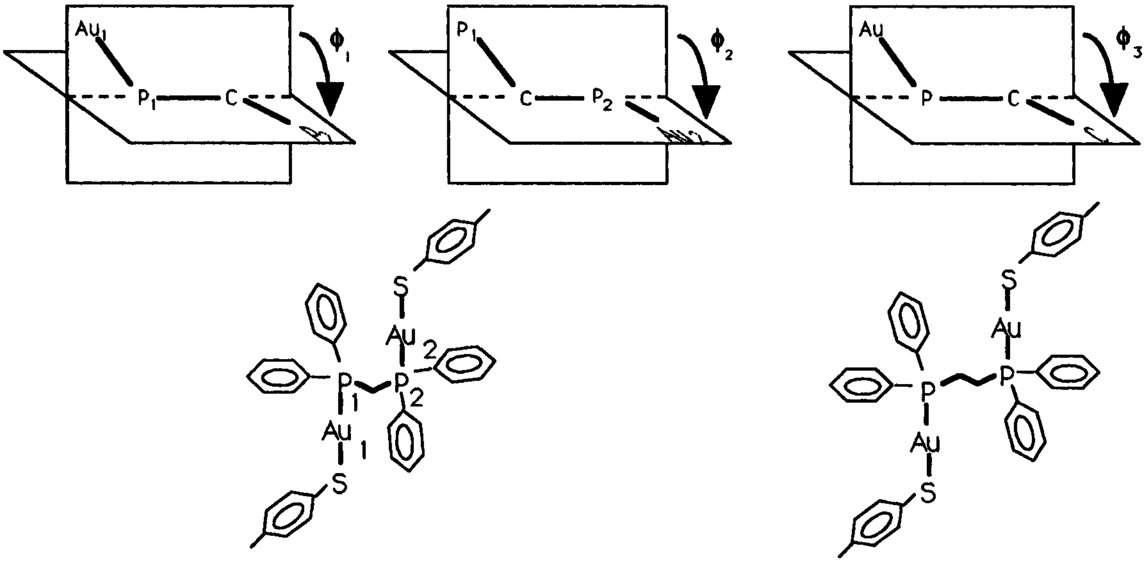

**Results and Discussion**

**Variable Temperature $^{31}P\{^1H\}$ NMR Experiments.** At room temperature, each of the complexes in series **A-C** exhibits a singlet in the $^{31}P\{^1H\}$ NMR spectrum between 28 and 37 ppm, a region that is typical for neutral, two-coordinate gold(I) complexes. Variable temperature NMR experiments on **A4**[5a] and **A2** reveal a singlet for each complex down to -80 °C, consistent with the equivalence of all phosphorus atoms in solution (Figure 2a). In contrast, complex **A1** shows two broad peaks at -80 °C at 30.1 and 32.2 ppm (Figure 2b).[5a] An activation energy of 10 kcal/mol was calculated from line shape analysis using a coalescence temperature of -60 °C and a peak separation at the low temperature limit of 170 Hz. Rotation about C-P single bonds can occur with activation energies near 10 kcal/mol.[11] However, the absence of a temperature dependent process for **A2** suggests that C-P bond rotation in the open-chain complexes (series **A**) have much lower activation energies. Molecular mechanics calculations were carried out to gain insight into the conformational preferences and barriers to rotation predicted for **A1** and **A2**.

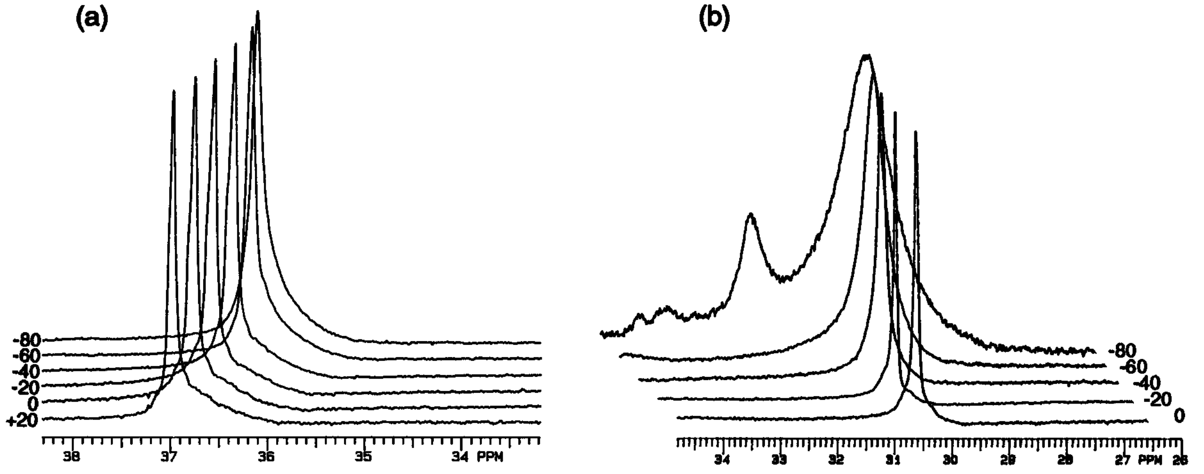

**Figure 2.** Variable temperature $^{31}P$ NMR spectra in $CD_2Cl_2$ from -80 to +20 °C as indicated: (a) $[Au_2(dppe)(p\text{-}tc)_2]$ (**A2**), no offset; (b) $[Au_2(dppm)(p\text{-}tc)_2]$ (**A1**), spectra offset for clarity.

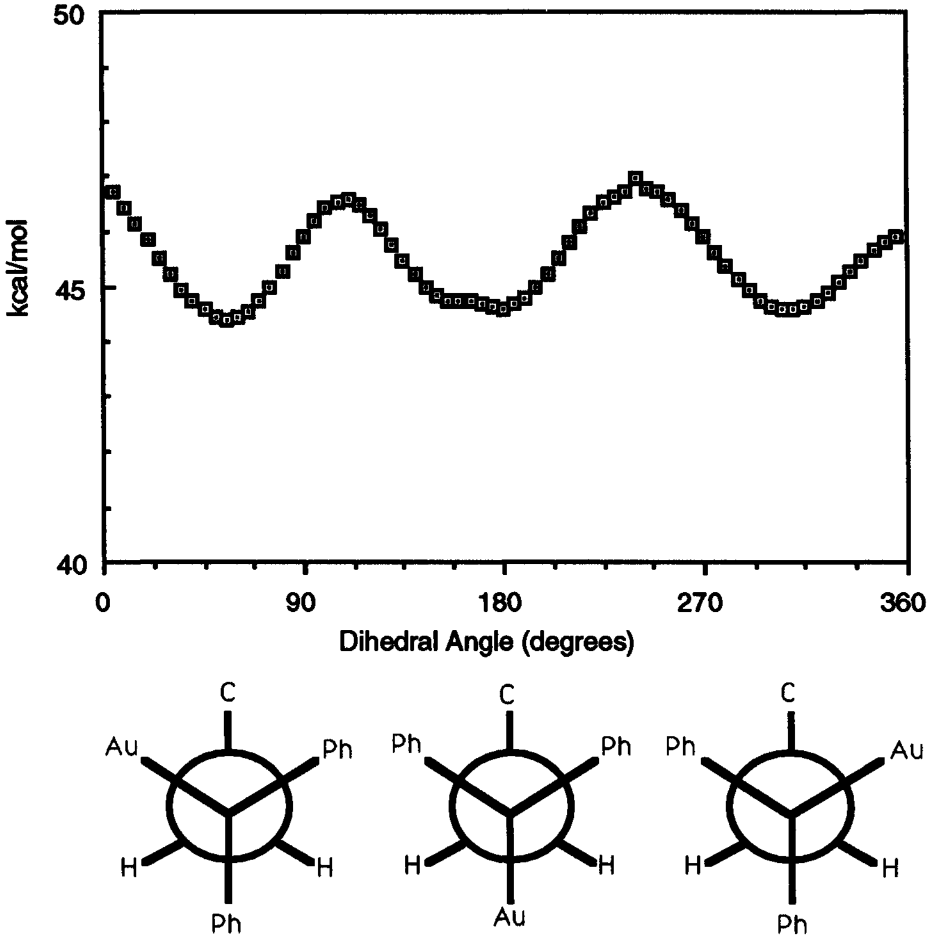

**Figure 3.** Conformational energy profile and Newman projections for staggered geometries of $[Au_2(dppe)(p\text{-}tc)_2]$ (**A2**).

**Conformational Analysis of $Au_2$(dppe)(*p*-tc)$_2$ (A2).** The conformational energy profile for **A2**, is shown in Figure 3. A series of maxima and minima, separated by only 2.5 kcal/mol, are observed for rotation about the C-P bond in **A2**. The maxima and minima occur every 120°, corresponding to approximately eclipsed and staggered conformations, respectively. The closest approach of gold atoms is greater than 4 Å. The barrier for rotation of **A2** is small compared to the available thermal energy at -80 °C. The molecular mechanics calculation is therefore consistent with VT-NMR experimental data that shows only one conformation of **A2** in the temperature range -80 to 20 °C.

**Conformational Analysis of $Au_2$(dppm)(*p*-tc)$_2$ (A1).** The conformational energy profile for **A1**, shown in Figure 4, is not symmetrical as is observed for **A2**. The large asymmetry in the energy profile can be attributed to the more severe intramolecular steric effects between phenyl rings in the dppm ligand compared to the dppe ligand. As a result, the 60° and 300° conformations are not equivalent in energy as might be expected by a view of simple Newman projections (see bottom of Figure 4). These Newman projections are misleading since they imply that the total energy depends only on the described dihedral ($\phi_1$), when in fact, other dihedrals are minimized to structures that do not necessarily correspond to the pathway connecting two fully enantiomeric structures.

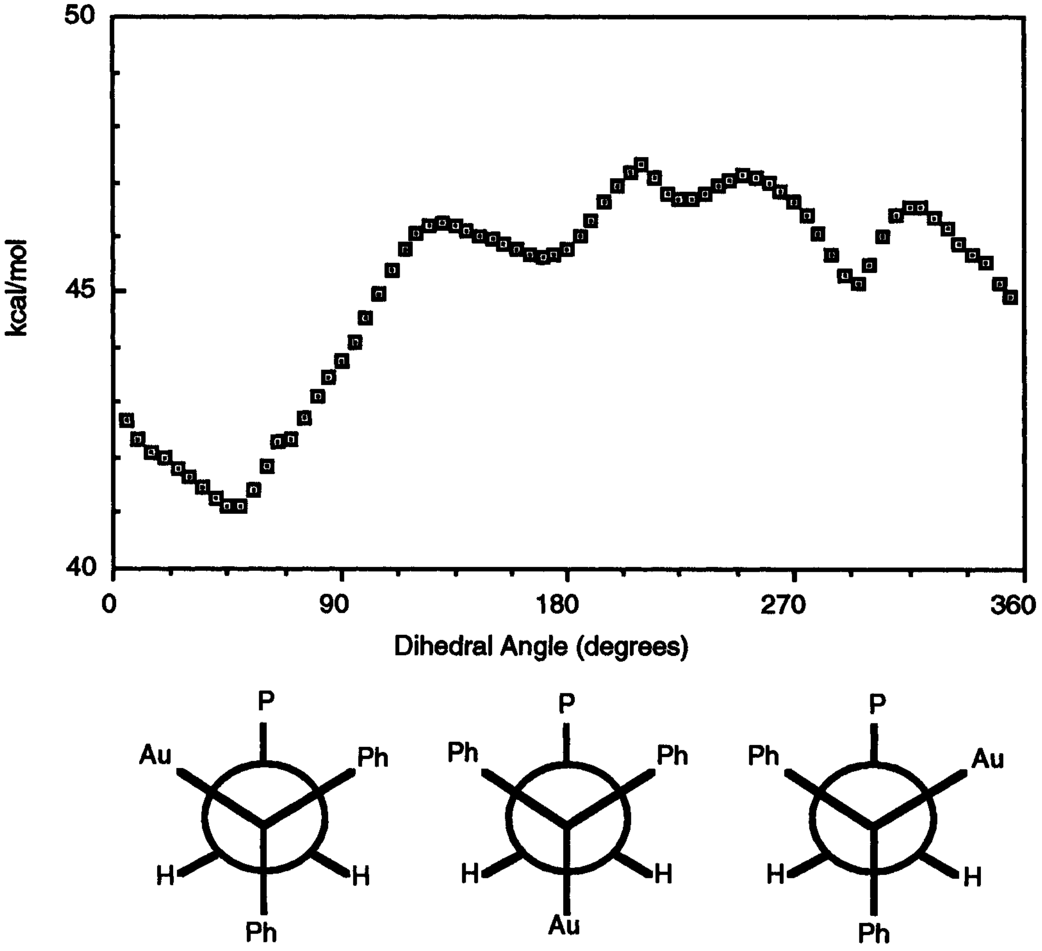

Figure 4. Conformational energy profile and Newman projections for staggered geometries of [$Au_2$(dppm)(*p*-tc)$_2$] (**A1**).

The unsymmetrical appearance of the energy profile prompted us to question whether a single dihedral drive computation of **A1** provided enough information about the accessibility of conformations. A more complete understanding of the conformational preferences can be obtained by a double dihedral drive calculation. (See Scheme I for definitions of $\phi_1$ and $\phi_2$.) The results for such a calculation, carried out on **A1** as described in the Computational Methodology section, are shown as a 3-dimensional plot in Figure 5.

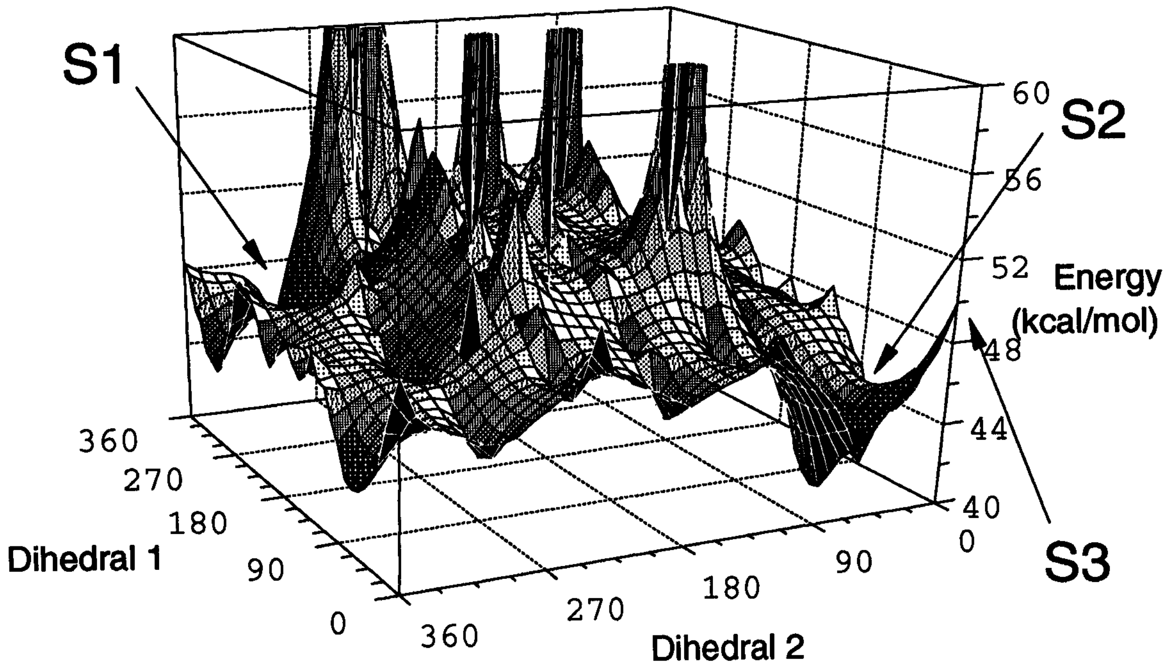

Figure 5. MMX 3-dimensional surface for $[Au_2(dppm)(p\text{-}tc)_2]$ (**A1**). S1 and S2 indicate enantiomers; S3 indicates a structure with the shortest distance between golds.

The 3-D plot is more informative than the 2-D conformational energy profile for the following reasons. First, the symmetry anticipated from the Newman projections but not seen in the 2-D conformational energy profile is now seen in the 3-D plot. The point labeled S2 in Figure 5 corresponds to the conformation near $\phi_1 \approx 60°$, i.e., the global minimum in the 2-D profile (Fig. 4). There is an equal energy conformation labeled S1 that corresponds to the enantiomer of S2. The 3-D plot also reveals a "mountainous" region where clashes between phenyl rings generate very large energy barriers to rotation. It is clearly impossible for S1 and S2 to interconvert along the diagonal. However, interconversion of the enantiomers can occur with an energy barrier as low as 6 kcal/mol by following a pathway closer to the edges of the 3-D surface.

This is more clearly seen in the contour plot shown in Figure 6. Structure S2 is represented by the region of lowest total energy shown in the lower left hand corner, and S1 is diagonally opposite. The energies of S1 (≈43.5 kcal/mol) and S2 (≈40.5 kcal/mol) appear to differ by 3.0 kcal/mol. This is an artifact of the way the double dihedral drive computation was carried out, i.e. $\phi_2$ was not driven in small enough increments. When the structures S1 and S2 are input as specified by $\phi_1$ and $\phi_2$ and full geometry minimizations are carried out, the energies differ by 0.2 kcal/mol. Ball and stick representations of S1 ($\phi_1 = 64°$, $\phi_2 = 59°$) and S2 ($\phi_1 = 299°$, $\phi_2 = 303°$) are shown in

Figure 7. The Au-P bonds in each structure are approximately "anti" to each other. We conclude that the enantiomeric structures, S1 and S2 represent the global minimum configuration. There are also several local minima along the surface connecting S1 and S2 that are within 3-4 kcal/mol of the global minimum. For example, the structure in the lower right hand corner of Figure 6 (near $\phi_1$ = 300°) corresponds to the local minimum conformation at 44.5 kcal/mol in the 2-D plot (Fig. 4).

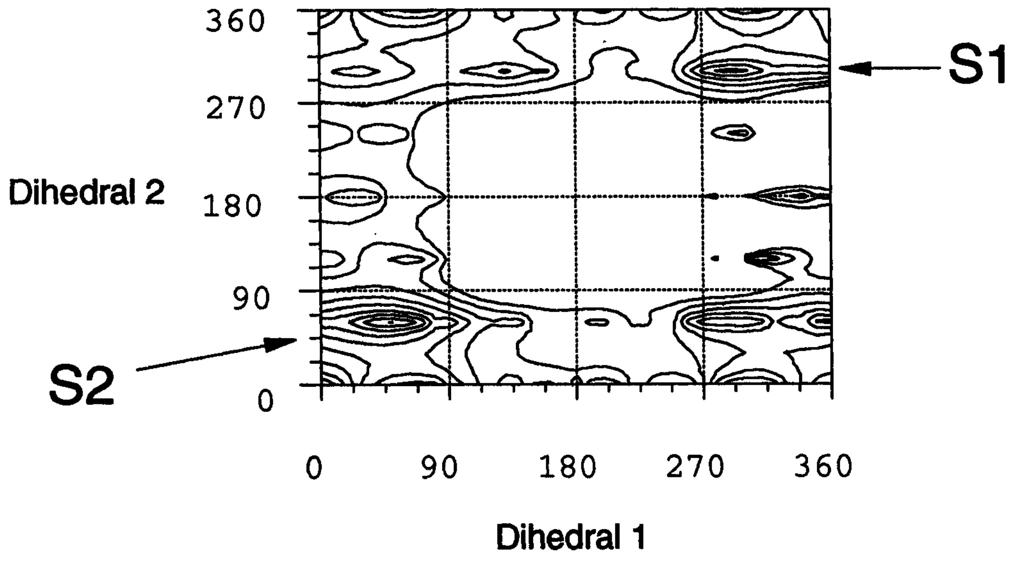

Figure 6. MMX contour plot for [$Au_2$(dppm)(*p*-tc)$_2$] (**A1**). Contour values of total energy were 40.5, 41.5, 42.5, 43.5, 44.5, 45.5, 46.5, 47.5, and 48.5 kcal/mol.

It is important to point out that interconversion of the global and local minima does not account for the dynamic process observed in the VT-NMR experiments for **A1** because the experimentally determined activation energy is 10 kcal/mol, while the activation barrier calculated by MMX is estimated to be no more than 6 kcal/mol.[12] Obviously, interconversion of S1 and S2 is also not responsible for the dynamic process because, as enantiomers, they would have the same chemical shift.

In a previous paper, we hypothesized that an intramolecular gold-gold interaction in **A1** causes repulsion between sulfur lone pairs and thus destabilizes the HOMO, which is sulfur in character.[5a] Does the molecular mechanics calculation support this hypothesis? There are a number of conformations in which the golds are within bonding distance (<3.4Å). However, the conformation which has the shortest distance between golds occurs at $\phi_1$ = 0°, $\phi_2$ = 0°, labeled S3 in Figure 5 and shown as a ball and stick representation in Figure 7. The Au-P bonds are oriented approximately "syn" to each other and the Au-Au distance is less than 3 Å. The MMX calculation does not include parameters for the attraction or repulsion between metal atoms. Accordingly, this conformation is disfavored for steric reasons and is estimated to be about 9 kcal/mol higher in energy than the global minimum. However, formation of a gold-gold bond in this conformation is expected to contribute electronic stabilization on the order of 8-10 kcal/mol.[13] The energy of the gold-gold bonded "syn" isomer would then be within 1 kcal/mol of the global minimum "anti" conformation. This interconversion of the "syn" gold-gold bonded and "anti" nonbonded conformations is consistent with the dynamic process observed in the VT-NMR experiments (see Scheme II).

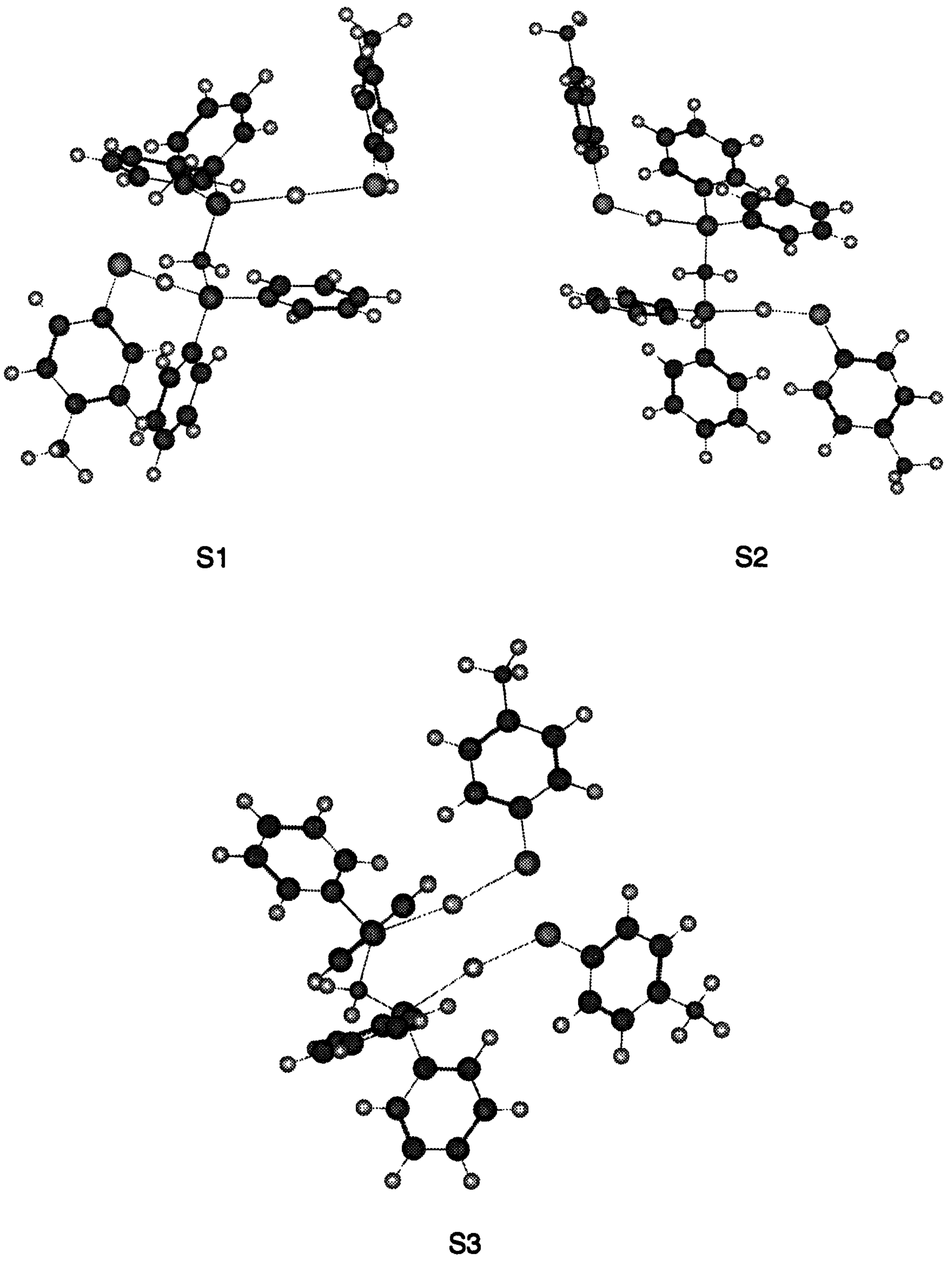

**Figure 7. Ball and stick representations of [$Au_2$(dppm)(*p*-tc)$_2$] (A1) in conformations S1, S2, and S3.**

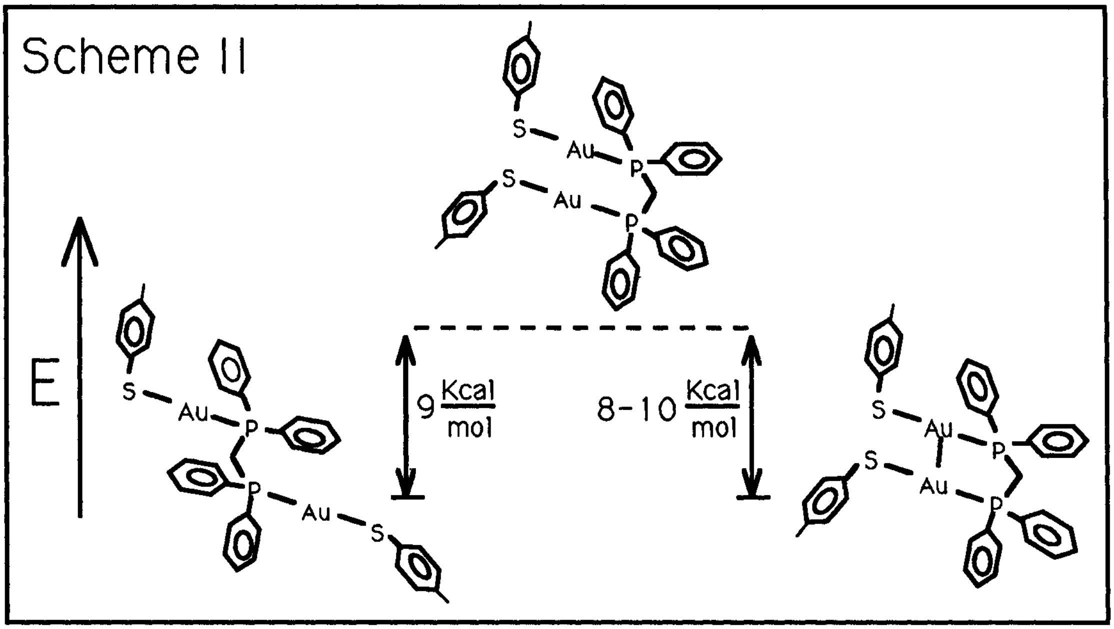

**The Effect of Sterically Hindered Thiolates on Gold-Gold Bonds.** Examination of the conformational profile of **A1** prompted us to investigate how bulky substituents on the thiolate aromatic ring would influence conformational preferences. The complexes shown below in series D were synthesized in which the steric and electronic properties of the substituents on the thiolate ligand are varied. We hypothesized that bulky substituents in the ortho positions would block the close approach of golds whereas meta or para substituents would exert less steric control. As a first approximation our hypothesis appears to be correct because the 2,6-disubstituted complexes are white and show only 1 peak in variable-temperature $^{31}P$ NMR experiments, while the 3,5-dimethyl and para-substituted derivatives are yellow and show 2 peaks at low temperature. We are continuing to investigate the electronic structure of these complexes to verify this conclusion and the results of electronic spectra analysis will be reported elsewhere.[14]

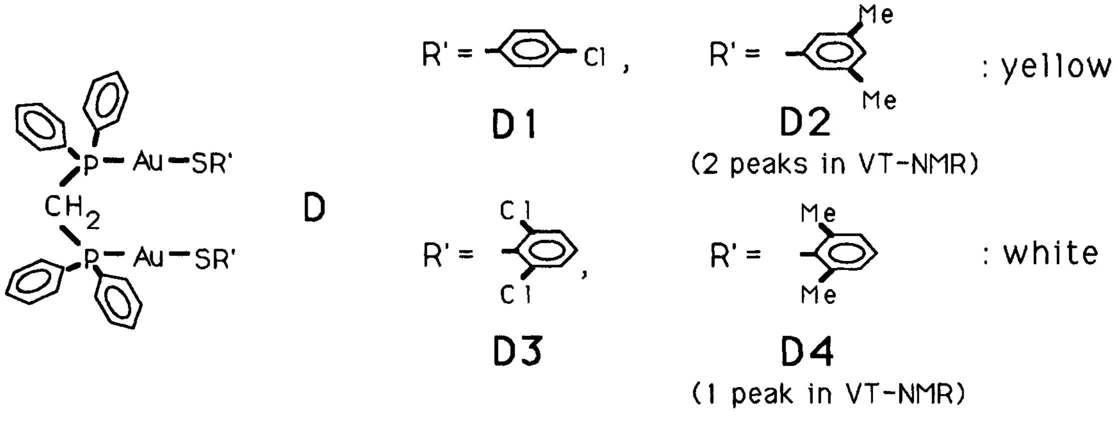

**Electronic Structure and Gold-Gold Interactions.** A principle piece of evidence for the weak attractive interactions between closed shell gold(I) atoms is provided by X-ray analysis of single crystals in which the distance between two gold atoms is significantly less than the van der Waal's radii of 3.4 Å.[3a] We have also recently reported for the first time the detection of gold(I)-gold(I) interactions using Extended X-ray Absorption Fine Structure (EXAFS) experiments conducted at the Stanford Synchrotron Radiation Laboratory by Professor R. C. Elder and his coworkers at the Univ. of Cincinnati.[5b] Gold-gold interactions are so prevalent in solid state structures of gold(I) complexes that the phenomenon has been dubbed "aurophilicity" by Schmidbaur.[3a] Furthermore, short gold(I)-gold(I) contacts are believed to be responsible for some unusual structural features. For example, the structure of the dinuclear complex **A4** reveals that the Au-P bonds are approximately anti to each other and the short *intermolecular* Au-Au distance of 3.094(1) Å leads to the formation of extended polymers.[5a] Schmidbaur and coworkers have recently reported several unusual structures in which "hypervalent" central atoms such as nitrogen or carbon are stabilized by gold-gold interactions.[15]

Establishing the presence of gold-gold interactions in solution is more difficult. We have used molecular mechanics calculations supported by VT-NMR experimental results to give us insight into the possibility of gold-gold bonding in solution. Molecular mechanics calculations have been successfully applied to conformational analyses of transition metal complexes in recent years.[16] Since force constants associated with Au(I)-P and Au(I)-S bonds are not readily available, the MMX force field uses a set of generalized force constants for transition metals. For this reason we have been careful to use the MMX calculations only for a qualitative understanding of the conformational preferences in **A1** and **A2**. The results of molecular mechanics calculations and VT-NMR experiments described above allow us to suggest an explanation for the red shift in the UV-vis spectrum of **A1** relative to **A2**. An intramolecular interaction between gold(I) atoms in the "syn" orientation would tend to increase repulsion between the lone pairs on sulfur. In addition to destabilizing the HOMO sulfur orbitals, a gold-gold interaction is expected to stabilize the LUMO if this empty metal orbital (probably 6p) contributes to the Au-Au $\sigma$ bond.[17] The net effect is a red shift in the S$\rightarrow$Au CT in **A1**. Although our studies are consistent with gold-gold bonds forming in solution for **A1** (and possibly for **D1** and **D2**), the results are not as yet definitive. Establishing whether this phenomena occurs in solution, and how it influences electronic structure and reactivity, is important because it may be critical to understanding the bioinorganic chemistry of gold.

We have recently done an interesting experiment that suggests that gold-gold bonding may influence solution reactivity. Reaction of complex **A1** and bis(*p*-chlorophenyl)disulfide occurs at room temperature and appears to proceed according to the equation shown below.[18]

$$Au_2(dppm)(SR)_2 + R'SSR' \rightarrow Au_2(dppm)(SR')_2 + RSSR$$

**A1** R = 4-methylphenyl, R' = 4-chlorophenyl

Formation of a significant amount of the symmetrical disulfide, RSSR suggests that a gold(I)-gold(I) bond in solution is mechanistically important. Oxidative addition of $X_2$ ($X_2$ = $CH_3I$, $PhCH_2Br$, $I_2$) to cyclic dinuclear Au(I) complexes with phosphorus ylide ligands generally leads to stable dinuclear Au(II)/(II) complexes.[19] "Anchimeric assistance" by an adjacent Au(I) may play an important role in these reactions.[20] By analogy, oxidative addition of R'SSR' may occur across two gold(I) centers, and if the dinuclear complex is in the "syn" orientation, then reductive elimination of RSSR could occur. Formation of RSSR is consistent with an oxidative addition/reductive elimination mechanism. Further experiments are underway to distinguish thermodynamic and kinetic products in the reactions of disulfides with mononuclear and dinuclear gold(I) complexes.

**Acknowledgments.** A.E.B. and M.R.M.B. gratefully acknowledge financial support from the National Institutes of Health (Grant AR39858) and the University of Maine BRSG funds. S. Ganesh and L. Lester are acknowledged for their technical assistance.

**References**

(1) (a) Walz, D. T. Mechanisms of Action of Gold Salts in Rheumatoid Arthritis. In *Advances in Inflammation Research*; Otterness, I., Capetola, R., Wong, S., Eds.; Raven Press: New York, 1984; Vol. **7**, 239. (b) Brown, D. H., Smith, W. E. *Chem. Soc. Rev.*, 1980, **9**, 217. (c) Shaw, C. F. *Inorg. Perspect. in Biol. and Med.*, 1979, **2**, 287. (d) Sadler, P. J. *Struct. Bonding (Berlin)* 1976, **29**, 171.

(2) (a) Mirabelli, C. K.; Hill, D. T.; Faucette, L. F.; McCabe, F. L.; Girard, G. R.; Bryan, L. B.; Sutton, B. M.; Bartus, J. O.; Crooke, S. T.; Johnson, R. K. *J. Med. Chem.*, 1987, **30**, 2181. (b) Okada, T.; Patterson, B. K.; Ye, S. Q.; Gurney, M. E. *Virology*, 1993, **192**, 631.

(3) for a few examples see (a)Schmidbaur, H. *Gold Bull.*, 1990, **23**, 11. (b) Pyykkö, P.; Zhao, Y. *Angew. Chem. Int. Ed. Engl.*, 1991, **30**, 604. (c) Balch, A. L.; Fung, E. Y.; Olmstead, M. M. *J. Am. Chem. Soc.*, 1990, **112**, 5181. (d) Raptis, R. G.; Fackler, J. P., Jr.; Murray, H. H.; Porter, L. C. *Inorg. Chem.*, 1989, **28**, 4057. (e) Jiang, Y.; Alvarez, S.; Hoffmann, R. *Inorg. Chem.*, 1985, **24**, 749. (f) Pyykko, P.; Desclaus, J. P. *Accts. Chem. Res.*, 1979, **12**, 276.

(4) Jaffey, D. M.; Madix, R. J. *J. Am. Chem. Soc.*, 1994, **116**, 3012.

(5) (a) Narayanswamy, R; Young, M. A.; Parkhurst, E.; Ouellette, M.; Kerr, M. E.; Ho, D. M.; Elder, R. C.; Bruce, A. E.; Bruce, M. R. M. *Inorg. Chem.*, 1993, **32**, 2202. (b) Jones, W. B., Yuan, J.; Narayanaswamy, R.; Young, M. A.; Elder, R. C.; Bruce, A. E.; Bruce, M. R. M. *Inorg. Chem.*, accepted for publication. (c) Turmel, C.; Jiang, T.; Wei, G.; Morrison, R.; Narayanaswamy, R.; Bruce, A. E.; Bruce, M. R. M. *J. Am. Chem. Soc.* (submitted). (d) Jiang, T.; Wei, G.; Turnmel, C.; Bruce, A. E.; Bruce, M. R. M. *Metal-Based Drugs*, accepted for publication.

(6) Lever, A. B. P. *Inorganic Electronic Spectroscopy*, 2nd ed.; Elsevier: Amsterdam, 1984.

(7) The UV-vis spectrum of **A2** is nearly identical to that of **A5**. Complex **A2** was selected for comparison to **A1** because the conformational analysis was simpler than for **A5**.

(8) Serena Software, Box 3076, Bloomington, IN 47402-3076; we thank Kevin Gilbert for a demonstration copy of this software.

(9) Burkert, U.; Allinger, N. L. *Miolecular Mechanics*, American Chemical Society, Washington, DC, 1982.

(10) Allinger, M. L.; Yuh, Y. H.; Lii, J.-H. *J. Am. Chem. Soc.*, 1989, **111**, 8551.

(11) Schmidbaur, H.; Deschler, U.; Milewski-Mahrla, B. *Chem. Ber.*, 1983, **116**, 1393.

(12) Conformational energies calculated by molecular mechanics are actually enthalpies. Even if there was a 1 kcal/mol entropic contribution to the activation barrier, this still would not account for a $\Delta G^{\ddagger}$ = 10 kcal/mol.

(13) Schmidbaur, H.; Graf, W.; Müller, G. *Angew. Chem. Int. Ed. Engl.*, 1988, **27**, 417.

(14) McDougal, K.; Bruce, A.; Bruce, M. manuscript in preparation.

(15) Dufour, M.; Schier, A.; Schmidbaur, H. *Organometallics*, 1993, **12**, 2408 and references therein.

(16) see for example (a) Mackie, S. C.; Baird, M. C. *Organometallics*, 1992,**11**, 3712. (b) Polowin, J.; Mackie, S. C.; Baird, M. C. *ibid*, 1992,**11**, 3724. (c) Hancock, R. D. in *Prog. in Inorg. Chem.*, 1989, **37**, 187. (d) Brubaker, G. R.; Johnson, D. W. *Coord. Chem. Rev.*, 1984, **53**, 1.

(17) Stabilization of the LUMO may occur if the 6s or 6p orbitals mix with the 5 $dz^2$ orbitals used to form a gold(I)-gold(I) interaction.

(18) Ganesh, S.; Bruce, A.; Bruce, M. unpublished results.

(19) (a) Murray, H. H.; Fackler, J. P., Jr.; Mazany, A. M. *Organometallics*, 1985, **4**, 154. (b) Basil, J. D.; Murray, H. H.; Fackler, J. P., Jr.; Tocher, J.; Mazany, A. M.; Trzcinska-Bancroft, B.; Knachel, H.; Dudis, D.; Delord, T. J.; Marler, D. O. *J. Am. Chem. Soc.*, 1985, **107**, 6908. (c) Fackler, J. P., Jr.; Murray, H. H.; Basil, J. D. *Organometallics*, 1984, **3**, 821. (d) Schmidbaur, H.; Franke, R. *Inorg. Chim. Acta*, 1975, **13**, 85.

(20) Collman, J. P.; Hegedus, L. S.; Norton, J. R.; Finke, R. G. *Principles and Applications of Organotransition Metal Chemistry*, University Science Books: Mill Valley, CA, 1987, p. 320.

